# Automated closed-loop continuous flow block copolymer synthesizer

**DOI:** 10.1039/d5sc07307c

**Published:** 2025-12-23

**Authors:** Wei Nian Wong, Daniel J. Phillips, Md Taifur Rahman, Tanja Junkers

**Affiliations:** a Polymer Reaction Design Group, School of Chemistry, Monash University 19 Rainforest Walk, Building 23 Clayton VIC 3800 Australia tanja.junkers@monash.edu; b Infineum International Ltd, Milton Hill Business and Technology Centre Abingdon OX13 6BD UK

## Abstract

A fully automated continuous flow synthesizer for diblock copolymer (BCP) synthesis was constructed comprising elements of flow chemistry, automation, machine learning and in-line monitoring. A new method using in-line FTIR spectroscopic analysis for accurate determination of monomer conversion (with an error as low as 2% relative to an NMR spectroscopic baseline) is presented, thereby generating a reliable feedback system for reaction self-optimisation using the platform. By employing reversible addition–fragmentation chain transfer (RAFT) polymerization at 100 °C, acrylates and acrylamides of different hydrophilicities (namely methyl acrylate, ethyl acrylate, butyl acrylate, 2-ethylhexyl acrylate, 2-hydroxyethyl acrylate, ethylene glycol methyl ether acrylate, diethylene glycol ethyl ether acrylate, 2-(dimethylamino)ethyl acrylate, acrylamide & *N*,*N*-dimethylacrylamide) were polymerized to make mixed BCPs, targeting different degrees of polymerization (15 to 100). Samples were collected automatically, and a BCP material library comprising 95 diblock copolymers (7 sets of double hydrophobic, 7 sets of amphiphilic and 3 sets of double hydrophilic monomer systems) with *M*_n_ ranging from 1800 g mol^−1^ to 14 700 g mol^−1^, was obtained in a high-throughput manner, with minimal human intervention throughout the entire process.

## Abstract 2

A fully automated continuous flow synthesizer for diblock copolymer (BCP) synthesis was constructed comprising elements of flow chemistry, automation, machine learning and in-line monitoring.

## Graphical abstract

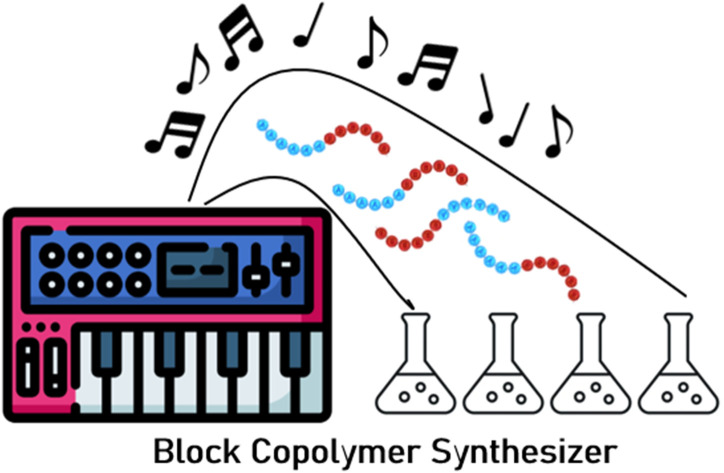

## Introduction

Block copolymers (BCPs) exhibit a broad variety of compositions and microstructures, making them an exciting class of adaptable material with many applications across the biomedical field,^1^ stimuli-response nanoparticles for drug delivery applications,^2^ nanofabrication for electronic applications,^3^ and membrane technology for environmental applications.^4^ Microphase separation in polymer self-assembly due to chemical dissimilarity of the individual blocks is also a unique characteristic that differentiates them from simpler polymers.^5^ The discovery of reversible deactivation radical polymerization (RDRP) has accelerated the synthesis of well-defined BCPs, and opened the door for the synthesis of polymers with increasingly complex architectures. Among the different RDRP techniques, reversible addition–fragmentation chain transfer (RAFT) polymerization holds a fortified position for block copolymer synthesis due to its high efficiency when operated under correct conditions, and its applicability to a broad range of monomers, solvents and process conditions.^6^

Despite the attractive properties of block copolymers, most industrial polymer applications are still dominated by conventional homopolymers and statistical copolymers, which is at least in part due to the significant hurdles associated with exploring new classes of materials. Even if RAFT polymerization is simple, it presents an increased cost and research & development burden. To challenge this status quo, there is a crucial need to streamline the material discovery process, and this is where flow chemistry and reaction automation can play a significant role. In comparison to batch chemistry, flow chemistry offers superior heat and mass transfer within a given reaction space, ease of reactor scale-up, high reproducibility of experimental results and inherently safer operability.^7,8^ These advantages make flow reactors an ideal platform with which to develop novel materials on scale in a cheaper and more time-efficient manner. Furthermore, the integration of real-time monitoring tools allows for rapid acceleration of research activity and enables high-throughput experimentation and analysis, providing the basis for powerful reaction automation. Examples of online monitoring techniques which find application in the polymer chemistry domain include nuclear magnetic resonance (NMR) spectroscopy,^9–12^ infrared (IR) spectroscopy,^13^ size exclusion chromatography (SEC)^14^ and electrospray ionization mass spectrometry (ESI-MS).^15^

Although the synthesis of block copolymers *via* a flow setup has been demonstrated before,^16–21^ a considerable amount of manual work is still required throughout the process, from varying the flow rate of a reaction stream to reaction sampling. For instance, Vandenbergh *et al.* synthesized various RAFT pentablock copolymers in a microchip reactor. However, each block required isolation and purification before subsequent chain-extension, introducing discontinuity in the process flow, potential error from human intervention and a notable increase in operation time.^20^ Hornung *et al.* utilized a commercially available flow system to produce block copolymers without the need for isolation. However, the flexibility of this approach is limited by the inability to change the volume of reactors and hence residence times in the second reactor.^16^ On the other hand, Perrier and co-workers constructed a looped flow reactor. By dosing monomers into the loop at different phases of the experiment, multiblock copolymers were successfully synthesized using just one tubular reactor.^22^ The same objective was achieved by Baeten *et al. via* a continuous multistage reactor cascade for high-throughput synthesis of multiblock copolymers. Although sophisticated, the aforementioned approaches could only produce one specific block copolymer per experiment, limiting their use as a high-throughput synthesis tool.^18^ Moreover, none of these reports integrates real-time monitoring into their system. Therefore, the volume of polymerization kinetic data collected was limited, and no automated data processing could occur, hence relying on constant human intervention to adapt reaction conditions. Generally, the integration of real-time monitoring tools into flow synthesis establishes an instant feedback system which enables autonomous closed-loop experimentation. In such systems, reaction parameter(s) can be improved iteratively by utilization of machine learning or other user-defined decision-making algorithms to satisfy a pre-defined objective function.^10,23,24^ The power of inline and online tools for the monitoring of polymerization kinetics has also been demonstrated. For instance, Van Herck *et al.* created a fully automated setup for real-time polymerization monitoring with in-line NMR spectroscopy and online SEC. The robustness of the approach was demonstrated by multiple users creating coherent datasets without prior training.^9,25^ Within the same group, Zhang *et al.* also demonstrated the application of inline IR spectroscopy for rapid screening of RAFT reaction parameters in a high-throughput manner.^13^ Rubens *et al.* were among the first to use online monitoring to achieve closed-loop experimentation in the domain of polymer chemistry, where a self-optimizing reactor was created to target different monomer conversions.^10^ By using similar analytical instruments, the same objective was also achieved by the Warren group, additionally introducing multiparameter Bayesian optimization to guide the reaction screening and optimization process.^23,26^

**Fig. 1:**
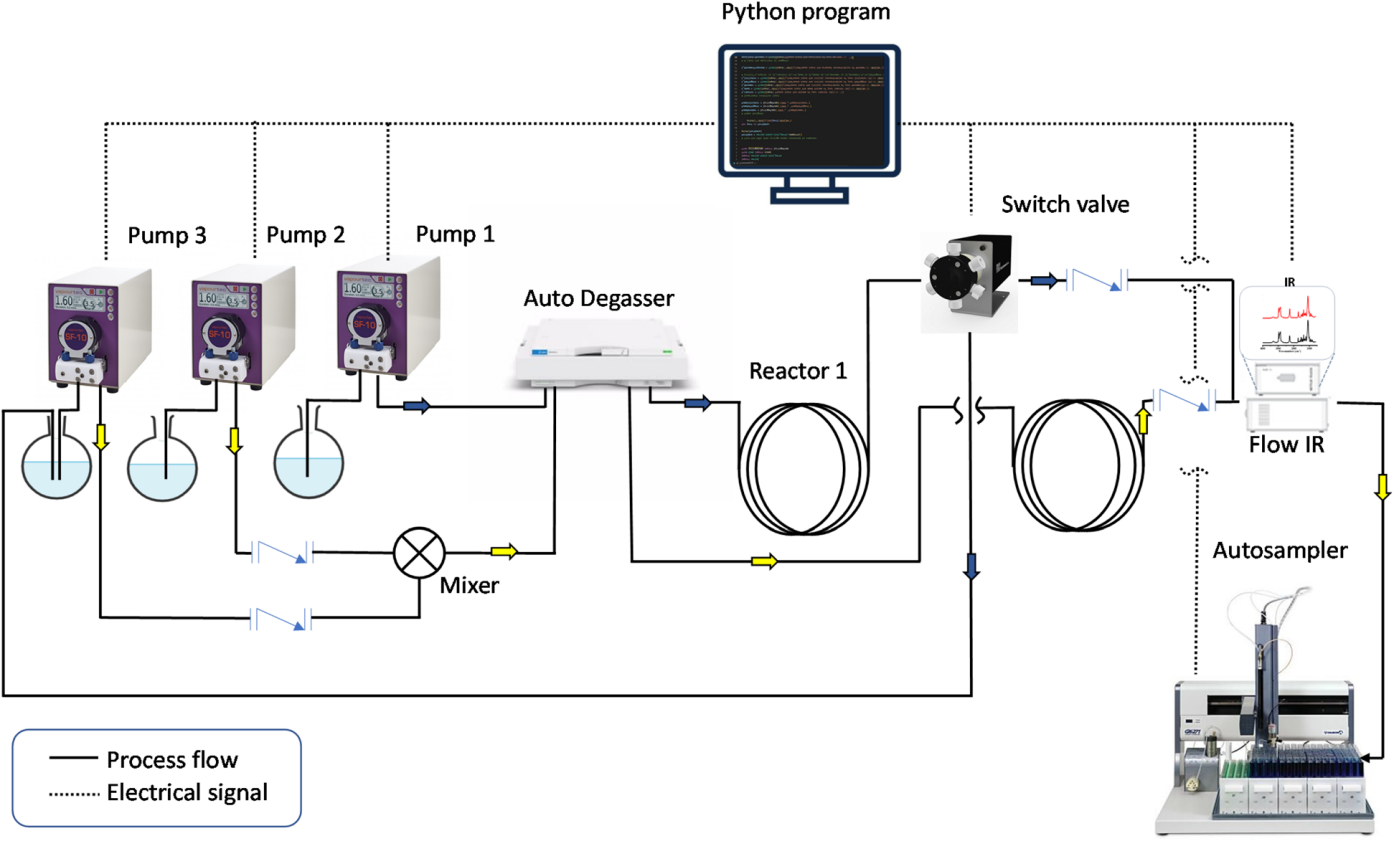
Schematic of the presented fully automated diblock copolymer synthesizer. Blue and yellow arrows indicate the process flow for the synthesis of homopolymer and diblock copolymer, respectively. The setup is constructed for two tubular reactors that are fully submerged in oil baths, three peristaltic pumps, an auto degasser, an FTIR spectroscopic monitor with a flow cell and a liquid-handling autosampler. Dotted lines indicate instruments that communicate with the Python program and are actively involved in the automation, while solid lines indicate the process streams.

While these closed-loop reactors are highly interesting for the production of individual polymers under specific conditions, approaches to the rapid production of wider functional sample libraries would further accelerate the development of new materials. To achieve this, a combination of self-optimization algorithms with robotic high-throughput experimentation is required. Herein, we describe such a combination, presenting a high-throughput, fully automated block copolymer synthesizer. To demonstrate its versatility, we utilized the system to construct a library of diblock copolymers combining a range of acrylate and acrylamide monomers, yielding polymers of a variety of chain lengths. With the concept of the “frugal twin” in mind,^27^ we constructed the setup with easily accessible lab tools that will allow similar machines to be installed elsewhere at reasonable cost. A schematic of the BCP synthesizer is outlined in Fig. 1. The machine comprises two reactor loops for homopolymer synthesis and successive chain extension, three peristaltic pumps to deliver reagents and solvent, and a robotic sample collector to store the obtained BCPs. To ensure continuous end-to-end operation of the machine and allow for self-optimization, a master Python program was written to control the hardware elements and to collect, process, and model kinetic data throughout the entire experiment. With the integration of in-line infrared spectroscopy, instant access to kinetic information is available throughout the experiment, which will be exploited by a decision-making algorithm to improve the process conditions autonomously. Finally, the synthesizer has not only the capacity to generate a diverse library of diblock copolymers, consisting of double hydrophilic, double hydrophobic and amphiphilic nature, but it also provides high-density kinetic data for each of the reactions, enabling future data driven applications.

## Experimental

### Reactor set-up

Two tubular reactors (3 ml for the first block and 3.4 ml for the second block), constructed from fluorinated gastight PFA tubing (1/16″ OD, 0.75 mm ID), were formed into loops and fully submerged in two mineral oil baths, which were heated to 100 °C on an IKA RCT hot plate. The volumes of both reactors was designed to be larger than those that were previously employed in our group,^10,18^ with the intention to reduce both the macro-RAFT agent collection time (first reactor) and the sampling time for each diblock copolymer (second reactor). Other passive volumes (where no reaction happens) that connected all the process units in the setup were introduced using tubing of the same material and dimensions, including a sampling loop (1.4 ml) for BCP collection before dispensing into vials by the autosampler. Three Vapourtec SF10 peristaltic pumps were used to control the flow rates of stock solution 1 (Pump 1), stock solution 2 (Pump 2) and macro-RAFT agent (Pump 3), respectively. A switch valve (Valco C4UWE Valve) was connected to the outlet of reactor 1 to direct its stream towards either (a) the IR flow cell or (b) a macro-RAFT reservoir. A Y-piece mixer (Y Assembly PEEK cd1/4-28.020 in) was used to merge macro-RAFT agent and stock solution 2 streams before passing into a degasser (Agilent 1260 Infinity). A liquid-handling autosampler (Gilson, GX-271) was connected to the outlet of the IR flow cell (Mettler Toledo ReactIR) for sampling of the polymers. Two check valves were placed downstream of both reactors to prevent backflow when either of them was directed toward the IR flow cell for kinetic monitoring. A stock solution 1 was prepared by mixing the monomer of choice, 2-(dodecylthiocarbonothioylthio) propionic acid (DoPAT) and 1,1′-azobis(isobutyronitrile) (AIBN) in butyl acetate, while stock solution 2 contained a different monomer and no RAFT agent (all details on parts used and further experimental details are found in the SI).

### Reactor process flow

The automated setup is fully controlled by a master Python script, and when initiated, the user is asked to input several parameters, including stock solution concentrations, desired target degree of polymerization (*DP*_Target_) for the first and second blocks, types of monomers involved, residence times for timesweep screening, and the target conversion for the first block. Other parameters like reactor or other non-reaction dimension (sampling volume, dead volume), IR scan interval *etc.* can also be changed (without prompt) when alterations to the setup are made (which, for example, may occur for maintenance reasons).

The process starts with the degassing of stock solution 1. This occurs *via* passing the reaction mixture into the auto-degasser unit for oxygen removal. Once the non-reaction volume is filled with deoxygenated reaction mixture, the program will then proceed to initiate a transient timesweep kinetic screening experiment according to the residence times inputted by the user. The timesweep screening experiment, made possible by the integration of an in-line monitoring tool, collects kinetic data during transient periods in the reaction (*i.e.* when the flow rate changes within the tubular reactor). Assuming the process operates in a plug flow regime, each plug is subjected to a different residence time, thus providing a comprehensive kinetic profile as the reactor ramps between the inputted start and end residence times.^9,28^ At the end of the timesweep experiment, all raw IR data is processed and the kinetic model exported in a comma-separated values (csv) file. After this initial fast screen, the setup then transitions automatically to a self-optimizing loop. Based on the timesweep data, a polymerization will be carried on the basis of the prediction made from the kinetic model. The monomer conversion value will then be compared to the target conversion, and the model updated if required. This cycle will repeat to fine-tune the kinetic model iteratively until the target is achieved. Upon achieving the the target conversion, the switch valve will direct the outlet of reactor 1 towards a macro-RAFT reservoir (connected to pump 3), and the setup will switch into macro-RAFT synthesis mode, at the optimal residence time. Afterwards, the setup will synthesize 15 ml of macro-RAFT before proceeding to the next step, to ensure a sufficient quantity is available for the next screening process.

The process flow for the second block is roughly the same as the first part of the Python script, except that two pumps (pump 2 and 3) are required to control the flow rates of both macro-RAFT agent and stock solution 2. An autosampler downstream of the IR detector will collect samples during the stabilization period of the timesweep experiment, which are later analysed by NMR and SEC for their monomer conversion and relative molar mass distributions (MMD). Details of the NMR and SEC analyses used for this study are available in the SI. The SEC system was calibrated using PMMA standards, and molar masses given are relative to these standards. These characterization results are complemented by the comprehensive kinetic data collected using IR spectroscopy throughout the experiment.

## Results and discussion

### Tracking monomer conversion by FTIR spectroscopy: determining an optimal wavenumber (WN) range for quantitative analysis

To use IR spectroscopy as an inline monitoring tool for reaction kinetic monitoring, we need to establish a calibration model for monomer consumption. This can be achieved by monitoring changes in IR frequencies associated with the monomer vinyl group. Peaks at 1630 cm^−1^ and 819 cm^−1^, which correspond to C=C bond stretching and twisting motions respectively, are suitable for this purpose; the former frequency is particularly preferable owing to its stronger absorption intensity and lower sensitivity to any fluctuation in ambient conditions.^29–31^ Multiple-point calibration models for a chemical system usually require frequent maintenance as they are prone to systematic errors due to fluctuations in chemical and physical characteristics of the chemical system and the analytical instrument. The deviation from Beer–Lambert's law necessitates the introduction of a correction coefficient to the evaluation. Additionally, the baseline drawn (between two wavenumbers) on the selected IR peak for integration has a significant impact on the obtained result and is always a subjective choice for different researchers.^32^

Using monomer conversion determined *via* NMR spectroscopy as a comparative benchmark, we applied a data science approach to determine a suitable IR wavenumber (WN) range, while maintaining good linearity with the Beer–Lambert law. To this end, the systematic screening of WN ranges between 1700 cm^−1^ and 1600 cm^−1^ was carried out. The approach performs max-min normalization with the IR spectra of the initial stock solution sample to account for fluctuations in ambient conditions that may affect the IR background. The optimal WN range for different acrylates and solvents was calculated based on polymerization samples collected in a prior experiment, and a Python algorithm was developed for the screening process to generate a heatmap, showcasing the discrepancy in values across different WN ranges. The WN range that showed the lowest error relative to the NMR spectroscopic baseline was chosen and implemented in the master Python program for automatic conversion determination.

**Fig. 2:**
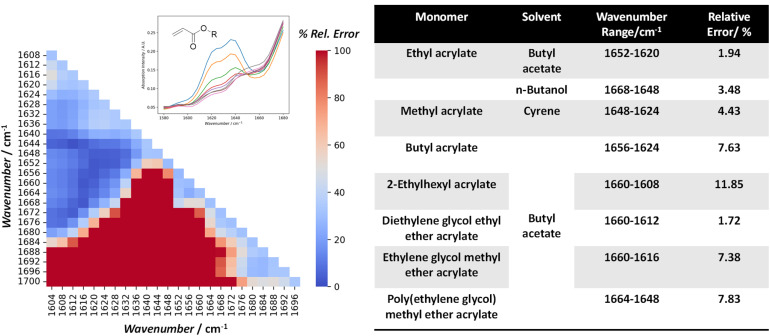
Heatmap of the relative error (in %), calculated based on the peak area integrated over different ranges of wavenumber on the IR spectra, from 1700 cm^−1^ to 1600 cm^−1^, and compared with the conversion values from NMR analysis, for all the collected. Table: optimal wavenumber range for quantitative analysis of residual monomers, covering a range of acrylic monomers in various solvents.

The left-hand side of Fig. 2 shows an example of such a heatmap, based on experimental samples collected from the RAFT polymerization of ethyl acrylate (monomer) in butyl acetate (solvent). The dark blue region highlights the WN range (1660–1612 cm^−1^) that showed the lowest error in measured conversion relative to NMR spectroscopic analysis, while the dark red region shows the highest error (>70%). 1652–1620 cm^−1^ was therefore selected for analysis as it shows the lowest error (1.92%), which is within the accuracy of a typical NMR spectroscopic experiment, and also comparable to the error range (<5%) demonstrated by previous work done within our group.^13^ A comparative study was also carried out using a predetermined IR calibration curve to quantify the monomer conversion for three different experiments (operated under different temperatures of 90 to 110 °C). Experimental samples were collected and analysed by NMR spectroscopy, and the average discrepancy was 6.68% (Table S4). The optimal WN range and the respective relative error for each of the acrylate monomers applied in this study under various solvents is shown on the right-hand side of Fig. 2. The error obtained *via* the FT-IR analysis is larger for acrylates like 2-EHA (11.85%), PEGMEA (7.83%), EGMEA (7.38%) and BA (7.63%), which could be due to errors in the NMR spectroscopic method used or due to insufficient calibration points in the FT-IR spectra. This could be, partly attributed to lower starting monomer concentration when bulkier monomers are used (1 M for PEGMEA and 3 M for EHA), and continues to decrease throughout polymerization. Consequently, a higher margin of error for FTIR analysis at such low concentrations is not uncommon.^13^ The details for homopolymerization conditions are provided in SI (Table S4). Among the other contributing factors are the interaction between monomers and solvents that can lead to shifts in spectral peaks^33^ and variations in the viscosity of reaction mixtures, which have a pronounced effect on the hydrodynamic flow profile within the reactor and sampling tube. On the other hand, it should be noted that only 4–5 samples were typically collected for NMR spectroscopic analysis, so the associated error could be reduced further by increasing the number of samples taken.

### Reaction design

In a typical multiblock reactor telescoping reaction, it is a requirement that each of the polymerization steps achieves full monomer conversion prior to chain extension in the subsequent unit. This is to avoid quasi-block copolymer formation due to incorporation of the residual monomer from the upstream unit into the second block.^34,35^ To maximize monomer conversion, the temperature was set to 100 °C. Despite the positive impact of high operating temperature on the polymerization rate, consideration has to be taken with respect to side reactions such as mid-chain radical formation^7^ and the initiator decomposition rate, which may lead to a ‘dead end’ polymerization scenario and limit the maximum monomer conversion that is theoretically achievable if the initiator depletes too quickly.^36,37^ Careful selection of the initiator concentration is also crucial because if it is too low, radical deficiency can lead to a low polymerization rate and/or limit monomer conversion. Conversely, too much initiator will eventually have a detrimental effect on the chain-end fidelity and dispersity of the synthesized polymer.^38^ A screening study on initiator concentration and temperature was carried out and it was decided that [M]_0_/[I]_0_ of 500 is the maximum allowable concentration before the control in polymerization deteriorates.^39^ For our purpose, all the stock solutions were prepared at [M]_0_/[I]_0_ = 750 to ensure good control over the diblock copolymerization. For DMAEA, we experimented with a higher initiator concentration ([M]_0_/[I]_0_ = 300), due to literature reports of its lower polymerization rate.^40^ To further enable maximum conversion, we leveraged understanding developed previously in our group, which showed that a residence time equivalent to 4–5 times the half-life of the exogenous initiator is optimal.^18^ This allows us to design a system that is applicable to all types of monomers being polymerized, as long as they are able to reach full conversion before the initiator is used up. A range of acrylates and acrylamides are used to build up the material library as they have higher rates of propagation compared with other vinyl monomers. Further optimization of the system is required before it can be applied for slower propagating monomers like methacrylates and styrenics; therefore, they are excluded in this study. Due to the limited solubility of RAFT agent in the reaction solvent, the starting monomer concentration of stock solution 1 was kept at 4 M, and to prevent reactor clogging, 2 M was chosen as the starting concentration for stock solution 2. On the other hand, PEGMEA_480_ and DEGEEA stock solutions were prepared at lower concentration (1 M and 3 M respectively) due to their bulkiness. A summary of all reaction parameters trialled is presented in Table 1.

**Table 1:** Homopolymerization and diblock copolymerization of various acrylates and acrylamides *via* DoPAT-mediated RAFT polymerization. Different DP_Target_ were targeted by adjusting the [M]_0_/[CTA], while the starting monomer concentration was varied according to the bulkiness of the monomer involved and its corresponding initiator concentration was set to maintain a consistent reaction rate

	Monomer	[M]_0_/M	[M]_0_/[I]_0_	DP_target_
First block	Ethyl acrylate (EA)	4	500	15–75
	Diethylene glycol ethyl ether acrylate (DEGEEA)	3		50
	Ethylene glycol methyl ether acrylate (EGMEA)	4		30
Second block	Methyl acrylate (MA), butyl acrylate (BA), 2-ethylhexyl acrylate (EHA), 2-hydroxyethyl acrylate (HEA), DEGEEA, acrylamide (AC), *N*,*N*-dimethylacrylamide (DMAC)	2	750	30–75
	PEGMEA_480_	1		30
	2-(Dimethylamino)ethyl acrylate (DMAEA)	2	300	30

### Homopolymerization: optimizing with timesweep kinetic screening

Based on the rationale outlined in the prior section, a timesweep kinetic screening experiment was carried out from a residence time (*t*_res_) of 2 min, to capture the kinetic profile at lower conversion region, up to 40 min, which is roughly 5 times the half-life of AIBN at 100 °C. In most cases, it was observed that the monomer conversion largely plateaus after *t*_res_ of 25 min (roughly 2 times the half-life of AIBN at 100 °C). Therefore, the timesweep screening was capped at 25 min, which also advantageously reduces experimental time. Details on the transient timesweep experiments are available in the literature.^9^ The Python algorithm that controls this section of the setup consists of two parts: (1) a flow rate-varying program and (2) a data slicing and processing program. The flow rate-varying program controls the peristaltic pump to which stock solution 1 is connected (Fig. 4a) and sets the flow rate according to residence time inputs by the user. By referring to the timestamp of the raw IR data, data slicing is carried out automatically to select only the data during the transient periods (shaded regions on Fig. 4b) of the experiment, and also (if needed), to isolate data during stabilization periods (circled regions on Fig. 4b) for cross-validation purposes with NMR spectroscopic analysis. Subsequently, vinyl peak integration and monomer conversion calculation will be performed and a kinetic model with conversion *vs. t*_res_ data (Fig. 4c) will be exported at the end of the experiment. The method to screen reactions only in the transient periods is established to deliver fast and reliable results, and indeed when data from the different transient sections is put together and recalculated for their respective reactor residence times, a smooth conversion *vs.* time plot is obtained (Fig. 4c). EA, EGMEA, DEGEEA and PEGMEA_480_ were chosen as the monomer for the first block due to their difference in hydrophilicity and molar mass and provide a useful point from which a wide range of diblock copolymers can be subsequently prepared. To vary the chain length of the first block, the monomer/RAFT agent ratio was altered, while all the other process conditions remained the same. The initiator to RAFT agent ratio was optimized to find a reasonable compromise between sufficiently high overall rate of polymerization and the fidelity of the obtained polymer (as expressed by the polymer dispersity). In the case of EA homopolymerization, the *DP*_Target_ was varied between 15 and 75. Both the observable rate of polymerization and maximum monomer conversion attainable (ranging from 89% to 94%) showed an increase with increasing *DP*_Target_, which is consistent with prior observations.^13^

**Fig. 3:**
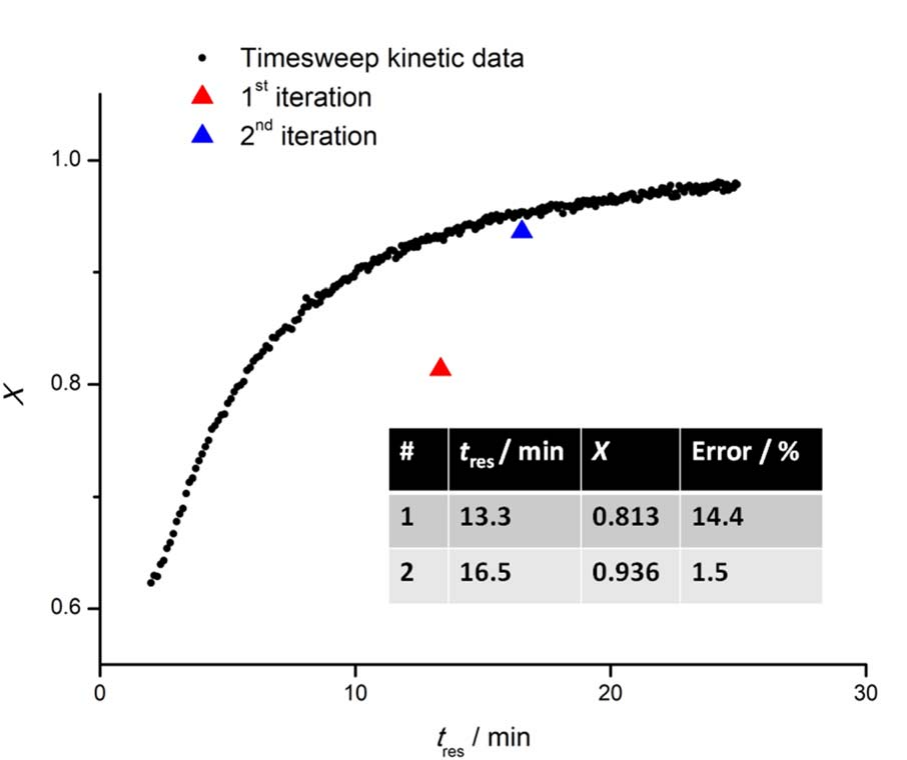
Timesweep experiment of EA (*DP*_Target_ = 30) across a range of 2–25 min, followed by a fine-tuning experiment in which target *X* (95%) was achieved in the second iteration, with a difference of less than or equal to 2%.

**Fig. 4:**
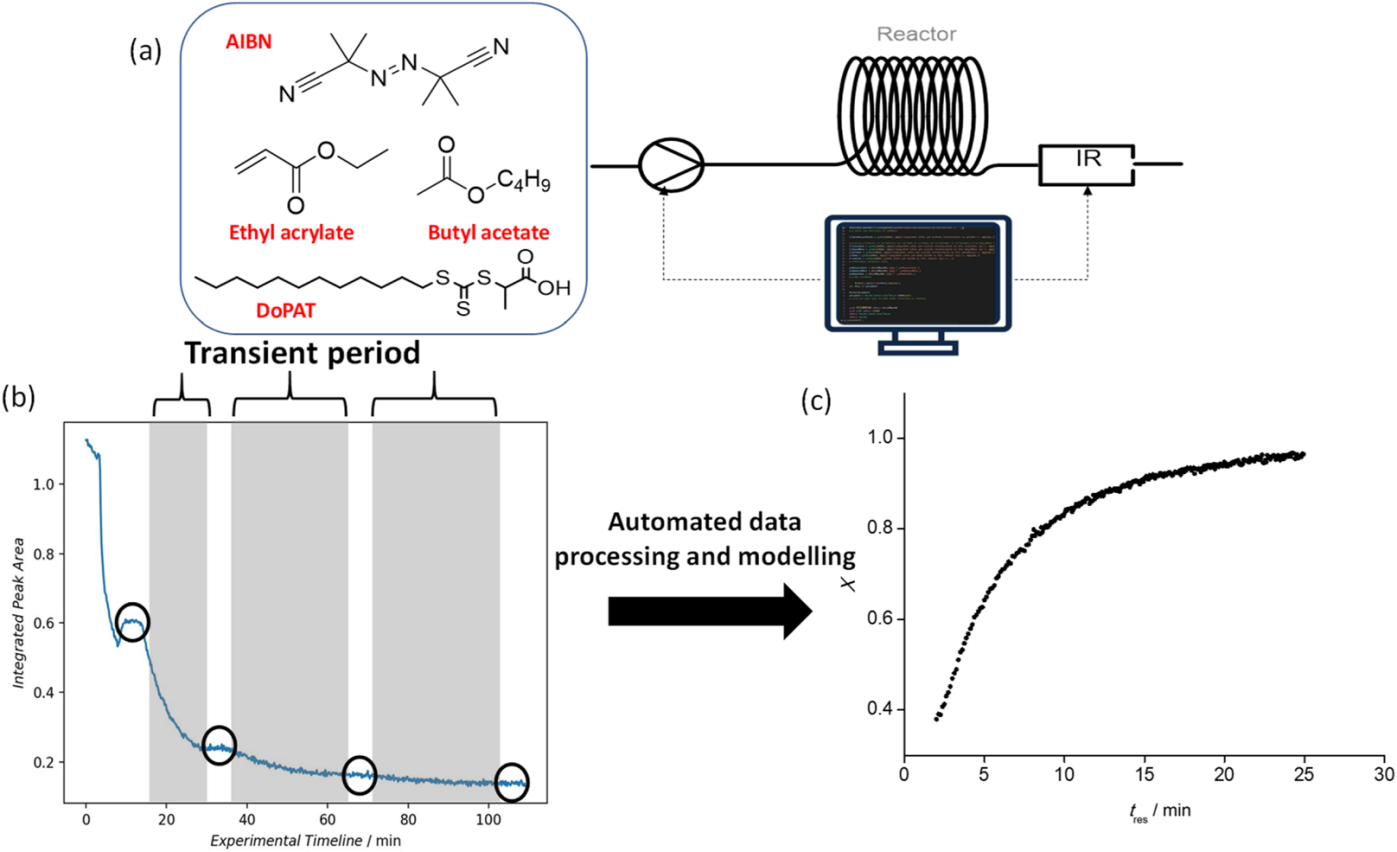
(a) Part of the automated setup for timesweep kinetic screening and self-optimising loop of homopolymerization. (b) Raw IR data collected and processed throughout a timesweep experiment of the polymerization of ethyl acrylate (EA). Circled region indicates stabilization period and sampling is carried out (optional), while shaded region indicates transient period. (c) Kinetic profile for the polymerization of EA, processed and exported from the Python program autonomously after the conclusion of timesweep experiment.

Next, the timesweep kinetic model obtained is used to predict the optimal residence time required for the reaction to reach a set monomer conversion, followed by iterative fine-tuning as needed (see below). This part of the process also serves as a corrective mechanism in the scenario where random errors (fluctuation in the ambient condition) or human errors (during preparation of the stock solution) are present, causing a discrepancy from the previously obtained kinetic model. For this purpose, whenever the same monomer is used as the first block, the user will be asked whether a timesweep experiment has been carried out before. If so, the data will be retrieved from a folder and used as a starting point from which the next experiment can be fine-tuned. To exemplify EA (Fig. 3), a new timesweep kinetic model for shorter *t*_res_ (2–25) min was obtained, and the maximum conversion achieved was approximately 97%. When this kinetic data was retrieved for use in a new experiment, it was regressed linearly using the Scikit-learn package in Python, with −ln(1 − *X*) as the independent variable, and *t*_res_ as the dependent variable. *t*_res_ of 13.3 min was first predicted, which resulted in *X* = 81.3%, a discrepancy of 14.4% relative to the target *X* (95%). The discrepancy could be due to various experimental factors like variation in the purity of chemicals used, difference in the ambient temperature or deviation of oil bath temperature from its setpoint. Moreover, the polymerization was assumed to follow first-order kinetics, but in reality, a deviation from linearity was observed due to depletion of initiator at very high monomer conversion.^41^ This shows that kinetics are only partially reproducible in a complex reactor setup due to outer influences. To tackle this, the self-optimising algorithm appended the latest data obtained to the previous dataset and assigned with an increased sample weight of 200. This strategy introduced a significant positive bias to the latest data collected. In this way, the resulting model was adjusted for the conditions in use. As exemplified in Fig. 4, a new *t*_res_ (16.5 min) was predicted by the updated model, resulting in *X* = 93.6%, a discrepancy of only 1.5% from the target, and less than the tolerable error margin (2%) set by the user. Hence, the experiment was deemed successful. In all fine-tuning attempts, the targets were achieved in the first or second iteration, highlighting the high accuracy and reproducibility of the timesweep approach. In the scenario where fine-tuning was carried out immediately after a timesweep experiment, the target conversion was achieved on the first iteration, as the reaction mixture was from the same source and the experimental errors outlined earlier were absent.

### Diblock copolymerization: mixing efficiency and its influence on chain extension

With the ability to make homopolymers established, we next turned our attention to the preparation of BCPs. BCP synthesis can only work well if the phases are mixed well. In a flow system, this is usually problematic. Generally, chain extension requires a low viscosity solution (monomer) to mix with a comparatively high viscosity solution (homopolymer). Mixing two streams of such phases is not trivial because (i) typical continuous flow laboratory setups fall within the laminar flow regime and thus mixing is predominantly diffusion-controlled, irrespective of the type of mixer used^42^ and (ii) the large molar mass of the macro-RAFT agent further impedes the mixing efficiency as its diffusion coefficient is exponentially smaller than other species in the reactor. We observed these limitations when trying to chain-extend macro-RAFT agents of varying molecular weight, from *DP*_Target_ 25 to 75 derived from ethyl acrylate (Fig. S4). Shoulders were observable on the low molecular weight side of the elugrams of PEA_50_-*b*-PBA_*x*_ and PEA_75_-*b*-PBA_*x*_, which overlapped with the macro-RAFT agents (PEA_50_ & PEA_75_) used in both cases. This indicates incomplete chain-extension of a significant amount of macro-RAFT agent in the reacting system. In diffusion terms, some of the macro-RAFT agent were not effectively mixed with the monomer, and hence these chains could not grow further. Moreover, when introducing a micromixer, which usually induces turbulent mixing, similar results were obtained (Fig. S5). However, PEA_25_-*b*-PEHA_*x*_ copolymers did not exhibit this issue, suggesting that improved homogeneity could be achieved with reduced molecular weight macro-RAFT agents. When swapping BA for MA, we found that co-polymers starting from a *DP*_Target_ PMA block of 50 chain-extended well, further validating the impact of the molecular weight contribution (MA having a lower molecular weight than EA).

Inspired by work from Chen and co-workers, and in an attempt to improve the chain extension of higher molecular weight macro-RAFT agents, we trialled the use of glass beads and an alumina-packed bed column downstream of the Y-mixer as a means to enhance turbulence.^43^ Although some improvement in the molar mass distribution of the resulting diblock copolymers was observed, a significant pressure drop occurred as a result of the increased volume now introduced to the reactor. We next tried to introduce a greater mixing time *via* diffusion by adding additional volume between the mixer and reactor.^21,34^ This approach solved the homogeneity issue, indicated by the absence of a low molar mass shoulder on the elugram of the synthesized diblock copolymers, but we saw a significant difference between the expected and obtained molar masses, suggesting the mixing between macro-RAFT agent and stock solution was still sub-optimal. We therefore turned our attention to other types of mixers and found that the use of both a static micromixer (Fig. S5a) and a T-mixer (Fig. S5b) led to a surprising decrease in molar mass with increasing conversion for PEA_50_-*b*-PMA_50_ BCPs. Since homogeneity didn't appear to be the cause (as indicated by the absence of the MMD shoulders discussed above), it was hypothesized that the deviation was due to an incorrect molar ratio of macro-RAFT agent to monomer in the reactor. This can occur when the individual flow rates differ significantly, resulting in a significant pressure gradient that partially impedes the flow of one stream. Thus, a Y-piece mixer was chosen as it offers the least resistance to the flow of the two streams. Further mixing was introduced by filling three channels of the auto-degasser with reagent prior to the timesweep experiment, under the same flow rates for both streams. This introduced sufficient reaction volume (3 × 12 ml) for the entire experiment, whilst ensuring consistency in the mixing ratio. When tested on the synthesis of PEA_50_-*b*-PBA_50_ BCP, close agreement between the apparent number-average molar mass (*M*^app^_n_), determined from SEC, and theoretical number-average molar mass (*M*^theo^_n_) was then satisfyingly observed (see Fig. S5c).

### Diblock copolymerization

Next, our experimental development progressed to stage 2 (chain extension), with the desired *DP*_target_ of the BCP inputted by the user. The macro-RAFT and monomer 2 stock solution streams are mixed prior to passing into reactor 2. The flow rate of the monomer in stock solution 2 is set again according to user specifications, and a timesweep experiment is completed in the same way as for block one. Monomer conversion at specified *t*_res_ was determined by NMR spectroscopy and matched with the vinyl peak area as measured by FTIR spectroscopy. This allowed an empirical equation to be established and removed the need to establish individual IR calibration models for each monomer introduced to the polymer. Moreover, the combination of offline and online characterization tools provides a comprehensive kinetic profile of the reaction and a more detailed characterization of the samples collected. Sample collection at different residence times (*t*_res_) was facilitated by a liquid-handling autosampler. Since samples are taken in the stabilization period between set timesweep experiments, a series of samples with growing second blocks is obtained automatically, and hence different *DP* for the second block can be obtained without the need for multiple repeats of the same experiment.

**Fig. 5:**
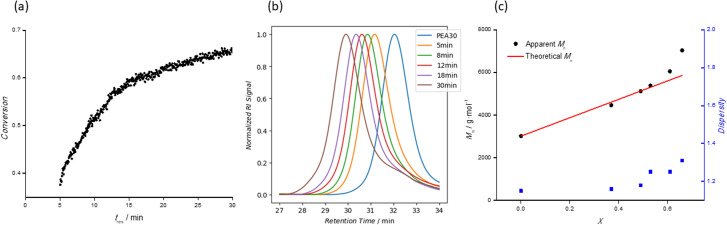
Data obtained from the synthesis of (PEA_30_-*b*-PMA_50_): (a) kinetic data from the timesweep experiment; (b) & (c) MMD and monomer conversion of PEA_30_-*b*-PMA_50_ diblock copolymers, collected at different residence times, upon chain extension of PEA_30_ homopolymer. Conversion ranging from 30 to 70% was achieved.

By way of example, Fig. 5 shows results from the preparation of a double hydrophobic BCP (PEA_30_-*b*-PMA_50_). Fig. 5a shows the plot of kinetic data (*t*_res_*vs. X*) obtained from the timesweep screening at *t*_res_ of 5–30 min. Conversions of up to 66% were observed in this case, lower than those observed during the homopolymerization of each monomer. The lower polymerization rates observed are consistent with literature reports,^34^ where the bulkiness (and hence slower diffusion rate) of the macro-RAFT agent impedes the overall rate of chain extension, thus requiring longer *t*_res_ to achieve monomer conversions comparable to a simple homopolymerization.^18^ Moreover, the lower initiator ([M]_0_/[I]_0_ = 750) and monomer concentrations ([M]_0_ = 1.33 M to 1.60 M) employed to both help maintain control of the diblock copolymerization whilst also preventing clogging of the reactor will also contribute to the slower reaction rate. Five samples were collected during the experiment, and the SEC traces of each are shown in Fig. 5b. The gradual shift of the SEC traces without any observable shoulder indicates successful chain-extension of the macro-RAFT PEA_50_, and the increase in MMD was consistent with increasing monomer conversion. Fig. 5c shows good agreement between *M*^theo^_n_ and *M*^app^_n_ plots, and *Đ* is less than 1.4 for all the BCPs, indicating good control of the polymerization.

### Diblock copolymer material library construction

To demonstrate the versatility and robustness of the setup, we utilized it to synthesize 37 double hydrophobic, 40 amphiphilic and 18 double hydrophilic BCPs. Firstly, an EA block was chain extended with hydrophobic monomers (MA, BA or EHA) or hydrophilic monomers (DEGEEA, HEA, DMAC, DMAEA or PEGMEA_480_) to form double hydrophobic or amphiphilic BCPs, respectively. To make double hydrophilic BCPs, an EGMEA block was extended with AC, DMAC or HEA. For each starting homopolymer block, a series of BCPs was collected using a liquid-handling autosampler. This strategy, in tandem with the application of different types of monomers and by varying *DP*_Target_ of the first block, enables the construction of a diverse BCP material library with minimal human intervention throughout the entire process flow (Table 2). This library ranged in *DP*_Target_ of the first block from 15–75 and the second block from 30–100, with *t*_res_ between 2–50 min.

**Table 2:** Molecular weight distributions of all the diblock copolymers synthesized by the automated setup, at 100 °C. *M*^theo^_n_ is calculated based on the conversion value from NMR spectroscopy. *M*^app^_n_ and *M*^app^_W_ are determined by SEC based on the Mark–Houwink parameters of PMMA. The average molar mass and monomer conversion for the first blocks are shown in the first row of each section, indicated by *t*_res_ = 0 min, while the subsequent rows show that for the diblock copolymers synthesized at different *t*_res_. Double hydrophobic, amphiphilic and double hydrophilic diblock copolymers are indicated by blue, red and green lines respectively

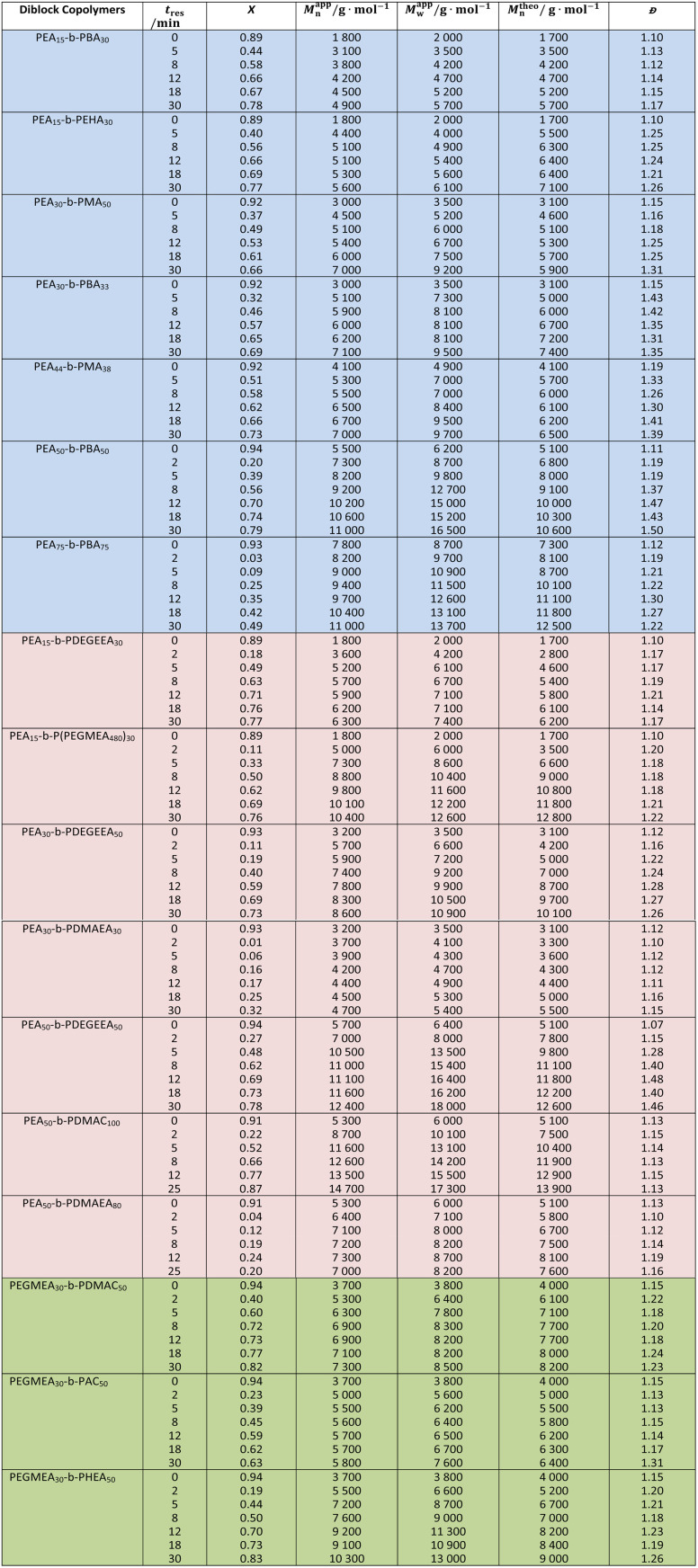

In this BCP library, monomer conversions ranged from 20–87% and molecular weights from 1800 g mol^−1^ to 14 700 g mol^−1^, depending on the *DP*_Target_ in the first and second block, and the choice of *t*_res_ (and its *X*). For instance, the copolymer set with the smallest molar mass (1800–4900 g mol^−1^) was PEA_15_-*b*-PBA_30_, and the largest (8700–14 700 g mol^−1^) was PEA_50_-*b*-PDMAC_100_. PEA_15_-*b*-P(PEGMEA_480_)_30_, showed a broader range of molar masses (5000–10 400 g mol^−1^) due to the large molecular weight of PEGMEA_480_ (*M*_n_ = 480 g mol^−1^). Narrow dispersity of all the BCPs synthesized (*Đ* < 1.5, and in most cases <1.3) indicates good control over the polymerization. It must be noted that the exact molar mass determination of BCPs using SEC is inherently difficult without absolute molar mass detectors. This is further exacerbated by discrepancies in solvation and miscibility properties between the individual blocks, especially when mixed hydrophobic and hydrophilic BCPs are assessed.^44^ As a result, the assumption of universal calibration in SEC does not hold true and the precise parameters required to use the Mark–Houwink–Sakurada (MHS) equation for each type of BCP are unavailable most of the time. Hence, a reasonable margin of error between the theoretical and apparent molar mass should be expected. Considering this, an average discrepancy of only 7.8% between theoretical and apparent *M*_n_ is reasonable.

When comparing polymerization kinetics for the monomers used, the hydrophilic monomers showed higher apparent polymerization rates, most notably with DMAC and HEA, where more than 80% of monomer conversion was attained in 30 min. This could be attributed to the higher polarity of the monomers and to hydrogen bonding between the monomers and polymer chain repeat units.^45,46^ When comparing hydrophilic acrylates to acrylamides, by using PEA_50_-*b*-PDEGEEA_50_ and PEA_50_-*b*-PDMAC_80_ as the examples, the monomer conversion achieved in both experiments is (27–78)% and (22–87)% respectively. However, AC, despite being the same monomer class as DMAC, showed a lower rate and lower monomer conversion (*X* = 63% at *t*_res_ = 30 min). This could be explained by a lower solubility of AC in the reaction solvent and may be resolved *via* the use of a more polar solvent like DMSO.^47^ In the synthesis of PEA_75_-*n*-PBA_75_ BCPs, lower overall conversion (*X* = 49% at *t*_res_ = 30 min) was observed. Rather than a systematic issue with the use of BA as a reactive monomer, this could be due to many experimental factors like fluctuations in the ambient temperature, inaccuracy in the temperature control of the hotplate being used, or impurities present in the chemicals used. DMAEA was the only monomer with a significantly lower polymerization rate, with a maximum *X* of 32 % at 30 min when used to chain extend a PEA_30_ homopolymer, and similarly low conversion when used to extend a PEA_50_ homopolymer. This could be due to the reactivity of DMAEA (as a tertiary amine) towards the thiocarbonyl group of the RAFT agent^13^ or its tendency to undergo self-catalysed hydrolysis of ester bonds in the side chains, and would be worthy of future study.^48^

The samples collected during homopolymerization of PEGMEA_480_ consistently showed a lower *M*^app^_n_ than its *M*^theo^_n_ (Fig. S6a). This is consistent with the literature, where branching in the polymer is reported to lead to a contraction in hydrodynamic volume and thus an underestimation of its apparent molar mass by SEC.^49,50^ When the same monomer was used for diblock copolymerization with a PEA_15_ homopolymer, *M*^app^_n_ > *M*^theo^_n_ at lower monomer conversion whilst the relationship inverts as more PEGMEA_480_ is incorporated (Fig. S6b). The use of EHA also saw significant molecular weight discrepancies, potentially due to the hydrodynamic volume of the hydrophobic alkyl branching group when measured by SEC using a DMF eluent (Fig. S7b). Indeed, when re-analysed by SEC using a tetrahydrofuran (THF) eluent (Fig. S7a), the *M*_n_ discrepancy reduced from 21% to 6.6%. Other individual copolymer samples that show a considerable *M*_n_ discrepancy (>15%) between their measured and apparent counterpart are PEA_30_-*b*-PMA_33_ (*t*_res_ = 30 min) and PEA_30_-*b*-PDEGEEA_50_ (*t*_res_ = 30 min). This could be the cumulative effect of errors in sample preparation for analysis, inaccuracy in molar mass determination from the SEC and/or the standard error observed with NMR spectroscopic analysis. PEA_44_-*b*-PMA_38,_ PEA_50_-*b*-PBA_50_ and PEA_50_-*b*-PDEGEEA_50_ exhibited *Đ* > 1.4. This was caused by targeting a larger *DP*_Target_ in both blocks. Thus, the [CTA]_0_/[I]_0_ will inevitably decrease (as the [M]_0_/[I]_0_ is fixed to maintain the same overall polymerization rate), and control over the polymerization is reduced.^18^ Furthermore, the bulkiness of monomer (DEGEEA), increased reaction mixture viscosity with higher *DP*_Target_ and monomer conversion in the second block, or random factors like fluctuation in ambient conditions (heat and light exposure in the laboratory) can lead to an impact of different extent on the control of the polymerizations. The latter factor is especially true as all experiments were carried out at different times of day or night, and each of them lasted more than 3 hours. Therefore, given the complexity and numerous factors affecting the control of polymerization, *Đ* < 1.5 demonstrates satisfactory control across all polymerizations performed in this study. A reproducibility study was carried out for the synthesis of PEA_50_-*b*-PBA_50_, conducted on three different days. The variance (in percentage) in the three sets of results was within the acceptable range (8% for the apparent rate constant), further exemplifying the potential of such system in accelerating material discovery. A detailed comparison of experimental runs for reproducibility elucidation is provided in SI (Fig. S8 and Table S6).

## Conclusion

A fully automated setup for the high-throughput synthesis of diblock copolymer libraries of varying polarities has been presented. This approach utilises in-line FTIR monitoring to optimize reaction kinetics in a data-centric manner, an in-line degasser to remove manual deoxygenation processes and an autosampling method to minimise operator involvement throughout the entire process flow. A practical complication caused by the need for effective mixing of viscous macro-RAFT agent and less viscous monomer streams was overcome by mixing directly through the degasser. The versatility of this approach was then demonstrated by constructing a diblock copolymer material library comprising 7 sets of double hydrophobic, 7 sets of amphiphilic and 3 sets of hydrophilic diblock copolymers (95 samples). In addition, with the integration of in-line FITR, 17 sets of kinetic models were obtained for homopolymerization and diblock copolymerization respectively, providing comprehensive kinetic insights for all of the involved reactions.

This approach allows an “on demand” means to access a broad material library, removing some of the repetitive synthesis tasks typically observed with batch polymerizations. In principle, the scope of this approach, though not tested in this report, could be expanded to collect products for extended times, allowing larger quantities of polymer (100 g or more) to be isolated.

Regardless, this automated reactor marks the full integration of self-driving lab principles and library synthesis for polymer discovery. In principle, after the user has specified their desired target polymer, the outlined reactor is able to run entirely by itself, with human interaction only required for the loading of monomers and RAFT agent, and the characterization of the residual polymers. The entire process from optimization of the first block synthesis (achieving high conversion to facilitate good block copolymer formation) to block extension and systematic sampling, is done by the synthesizer. The versatility of the system also means expansion with additional analytical instruments, pumps or reactors is possible, and it can be modified easily to serve different research purposes. For instance, *via* the addition of online SEC and pumps to control the monomer and RAFT agent flow rates individually, the system can be transformed into a self-driving lab for molecular weight targeting of the first and second block, by using the same optimization logic as the monomer conversion targeting that we have used in this study. A light source can also be integrated into the reactor setup for photopolymerization of slower propagating monomers, making polymerization of monomers such as methacrylates, styrene or others feasible.^51^ As such, this system marks an important step towards machines that can carry out complex polymer synthesis in a truly autonomous fashion.

## Author contributions

Wei-Nian Wong: conceptualization, methodology, visualization, investigation, validation, and writing – original draft. Daniel J. Phillips: writing – reviewing and editing and supervision. Md Taifur Rahman: writing – reviewing and editing and supervision. Tanja Junkers: conceptualization, methodology, writing – reviewing and editing and supervision.

## Conflicts of interest

The authors declare no conflict of interest.

## Supplementary Material

SC-OLF-D5SC07307C-s001

## Data Availability

The data that support the findings of this study are available in the Monash research repository https://doi.org/10.26180/29396444. Supplementary information (SI): details of synthetic procedures, reactor setup, characterization techniques and additional results. See DOI: https://doi.org/10.1039/d5sc07307c.
