# Supplementary material for: Automated closed-loop continuous flow block copolymer synthesizer
Source: Chem Sci. 2025 Dec 23;17(8):4222–34. doi: 10.1039/d5sc07307c (PMC12784417; doi:10.1039/d5sc07307c)
Supplement: SC-017-D5SC07307C-s001 [file SC-017-D5SC07307C-s001.pdf]

Supplementary Information (SI) for Chemical Science.
This journal is © The Royal Society of Chemistry 2025

Supporting Information

Automated Closed-Loop Continuous Flow Block Copolymer Synthesizer

Wei Nian Wong,[a] Daniel J. Phillips[b], Md Taifur Rahman,[b] and Tanja Junkers *[a]

[a] Polymer Reaction Design Group, School of Chemistry, Monash University, 19 Rainforest Walk, Building 23, Clayton, VIC 3800, Australia. Email: tanja.junkers@monash.edu

[b]Infinuem International Ltd., Milton Hill Business and Technology Centre, Abingdon, OX13 6BD, United Kingdom.

Contents

Contents 1
Materials 2
Experimental 2
Stock solution preparation 2
Reactor Setup 2
Computation for flow rates 3
Optimal wavenumber range for quantitative analysis of residual monomer 4
Mechanism for thermal-initiated RAFT diblock copolymerization 5
Characterization 5
High-field nuclear magnetic resonance (H-NMR) spectroscopy 5
Size Exclusion Chromatography (SEC) 6
Fourier Transform Infrared (FTIR) spectroscopy 6
Logic for self-optimizing algorithm 7
Supplementary results 7
Effect of temperature, initiator concentration and residence time on monomer conversion 8
Effect of macroRAFT molecular weight on the chain extension efficiency 9
Type of mixer and influence on the chain-extension 10
Homopolymerization and Diblock Copolymerization with $PEGMEA_{480}$ 10
References 12

Materials

1,1'-azobis(isobutyronitrile) (AIBN) (Sigma-Aldrich, 98%) was recrystallized twice from methanol prior to use. 2-(dodecylthiocarbonothioylthio)propionic acid (DoPAT)) was synthesized according to a literature procedure.[1] N-Butyl acrylate (nBA) (Sigma Aldrich, ≥99%), methyl acrylate (MA) (Sigma Aldrich, 99%), ethyl acrylate (EA)) (Sigma Aldrich, ≥99.5%), 2-hydroxyethyl acrylate (HEA)) (Sigma Aldrich, 96%), 2-ethylhexyl acrylate (EHA) (Sigma Aldrich, 98%), di(ethylene glycol) ethyl ether acrylate (DEGEEA) (Sigma Aldrich, ≥85%), poly(ethylene glycol) methyl ether acrylate (PEGMEA) (Sigma Aldrich, M_n 480), ethylene glycol methyl ether acrylate (EGMEA) (Sigma Aldrich, 97.5%), dimethylaminoethyl acrylate (DMAEA) (Sigma Aldrich, 98%), acrylamide, N, N-dimethylacrylamide (DMAC) (Sigma Aldrich, 99%), cyrene (Sigma Aldrich, 98.5%), butyl acetate (Sigma Aldrich, 99.5%), dimethyl sulfoxide(Sigma Aldrich, 99.9%), n-butanol (Sigma Aldrich ,99.85%) were used as received.

Experimental

Stock solution preparation

In a typical procedure, a homopolymerization stock solution with a monomer concentration of 4M, which contains the RAFT agent DoPAT (5.7mmol, 1 eq.), the thermal initiator AIBN (0.57mmol, 0.1 eq.), ethyl acrylate as the monomer (0.285mol, 50 eq.) and butyl acetate as the solvent (42.43ml), is prepared and kept in sealed Duran flasks, connected to the set-up via peristaltic pumps (SF10, Vaportec). Target DP is adjusted by varying the ratio of [monomer]/[DoPAT] to 15, 30, 50 and 75.

In a typical procedure, a diblock copolymerization stock solution with a monomer concentration of 2M, which contains monomer that forms the second block (0.06mol, 1 eq.) and thermal initiator AIBN (0.08mmol, 0.0013 eq.), and butyl acetate as the solvent (24.56ml), is prepared and kept in sealed Duran flasks, connected to the set-up via peristaltic pumps (SF10, Vaportec). The calculation for stock solution preparation is outlined in Table S1 (homopolymerization) and Table S2 (diblock copolymerization) respectively.

Table S1: Composition of stock solution for homopolymerization.

Species	Raw Material	Density (g/ml)	Molecular Weight (g/mol)	Mole(mol)	Mass(g)	Molar Equiv	Volume (ml)
RAFT Agent	DoPAT		350.6	0.005704507	2.00	1.00	0.00
Monomer 1	Ethyl Acrylate	0.94	100.12	0.285225328	28.56	50.00	30.38
Initiator	AIBN		164.21	0.000570451	0.09	0.10	0.00
Solvent	Butyl Acetate						42.43
						Total Volume	72.81

Table S2: Composition of stock solution for diblock copolymerization.

Species	Raw Material	Density (g/ml)	Molecular Weight (g/mol)	Mole(mol)	Mass(g)	Molar Equiv	Volume (ml)
Monomer 2	Methyl Acrylate	0.95	86.09	0.06	5.1654	1	5.437263158
Initiator	AIBN		164.21	0.00008	0.0131368	0.001333333	0
Solvent	Butyl Acetate						24.56273684
Standard (NMR)	DMF						A few drops
						Total Volume	30

Reactor Setup

Table S3: Overview of flow parts used in setup.

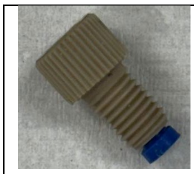	IDEX XP-230	Flangeless Fitting Natural, PEEK, 1/4- 28 Flat-Bottom, for 1/16" OD
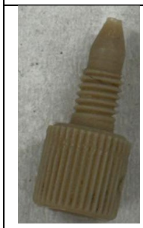	DEX F-120	One-Piece Finger tight 10-32 Coned, for 1/16" OD Natural

Classified as Confidential

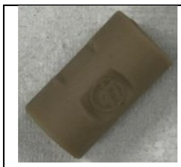	IDEX P-702	Union Assembly PEEK .020 thru-hole, for 1/16" OD"
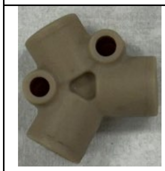	IDEX P-512	Y Assembly PEEK 1/4-28 .020in
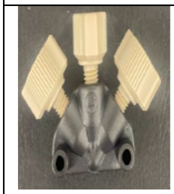	IDEX U-466	High Pressure Mixing Tee UHMWPE Frit 10um
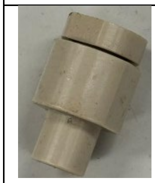	CV-3340	Check Valve Inline Non-Metallic 10-32
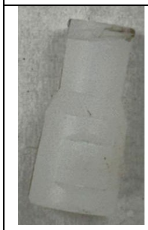	IDEX P-627	Threaded Adapter with Fitting, PEEK, 0.020" ID, 10- 32 Coned (F) to 1/4-28 Flat Bottom (F)

Computation for flow rates

$$\boldsymbol{F}_{\mathbf{tot}} = \frac{\boldsymbol{V}_{\mathbf{reactor,2}}}{\boldsymbol{T}_{\mathbf{res}}} \quad \mathbf{(1)}$$

$$\boldsymbol{F}_{\mathbf{tot}} = \boldsymbol{F}_{\mathbf{macroRAFT}} + \boldsymbol{F}_{\mathbf{stock,2}} \quad \mathbf{(2)}$$

$$\boldsymbol{DP}_{\mathrm{Target,2}} = \frac{\boldsymbol{F}_{\mathbf{stock,2}}[\mathbf{Monomer\ 2}]}{\boldsymbol{F}_{\mathbf{macroRAFT}}[\mathbf{macroRAFT}]} \quad \mathbf{(3)}$$

With user defined values of $V_{\mathrm{reactor,2}}$, T_{res}, $DP_{\mathrm{Target,2}}$, [Monomer 2], [macroRAFT]:
Solve **(1)**, **(2)** & **(3)**:

$$\boldsymbol{F}_{stock,2} = \frac{\boldsymbol{F}_{\mathbf{tot}}\boldsymbol{DP}_{\mathbf{Target,2}}[\mathbf{macroRAFT}]}{[\mathbf{Monomer\ 2}] + \boldsymbol{DP}_{\mathbf{Target,2}}[\mathbf{macroRAFT}]} \quad \mathbf{(4)}$$

$$\boldsymbol{F}_{\mathbf{macroRAFT}} = \boldsymbol{F}_{\mathbf{tot}} - \boldsymbol{F}_{\mathbf{stock,2}} \quad \mathbf{(5)}$$

Where:
$\boldsymbol{F}_{\mathbf{tot}}$: combinatorial flow rate of both streams
$\boldsymbol{F}_{\mathbf{stock,2}}$: Flow rate of stock solution 2
$\boldsymbol{F}_{\mathbf{macroRAFT}}$: Flow rate of macroRAFT agent solution
[**Monomer 2**]: Concentration of monomer 2 in stock solution 2

Classified as Confidential

$[\mathbf{macroRAFT}]$: Concentration of macroRAFT in solution
$\boldsymbol{DP}_{\mathbf{Target,2}}$: Target degree of polymerization for second block.

Optimal wavenumber range for quantitative analysis of residual monomer

In a typical experiment, a stock solution for homopolymerization similar to Table S1 is prepared and kept in sealed Duran flasks. Due to the bulkiness of the monomer involved, the starting monomer concentration and its corresponding initiator concentration was set to maintain a consistent reaction rate (Table S4). Homopolymerization is carried out in a flow reactor for different residence times, with sampling carried in an automated manner during the stabilization period. The samples are then analyzed with NMR spectroscopy to calculate the residual monomer concentration and thus monomer conversion. The NMR spectroscopic result will be used in tandem with the IR spectra collected during the stabilization (sampling) period, for wavenumber range screening in the vinyl peak region (1700-1600cm^{-1}).,

Table S4 : Homopolymerization of various acrylates via DoPAT-mediated RAFT polymerization.

Monomer	Solvent	$[M]_0$	$[M]_0$ / $[I]_0$ / $[CTA]_0$
Ethyl acrylate	Butyl acetate	4	500 / 1 / 10
	n-Butanol	4	500 / 1 / 10
Methyl acrylate	Cyrene	4	300 / 1 / 10
Butyl acrylate	Butyl acetate	4	500 / 1 / 10
2-Ethylhexyl acrylate	Butyl acetate	3	600 / 1 / 12
Di (ethylene glycol) ethyl ether acrylate	Butyl acetate	3	500 / 1 / 10
ethylene glycol methyl ether acrylate	Butyl acetate	4	500 / 1 / 10
Poly (ethylene glycol methyl ether acrylate	Butyl acetate	1	300 / 1 / 10

Classified as Confidential

Mechanism for thermal-initiated RAFT diblock copolymerization

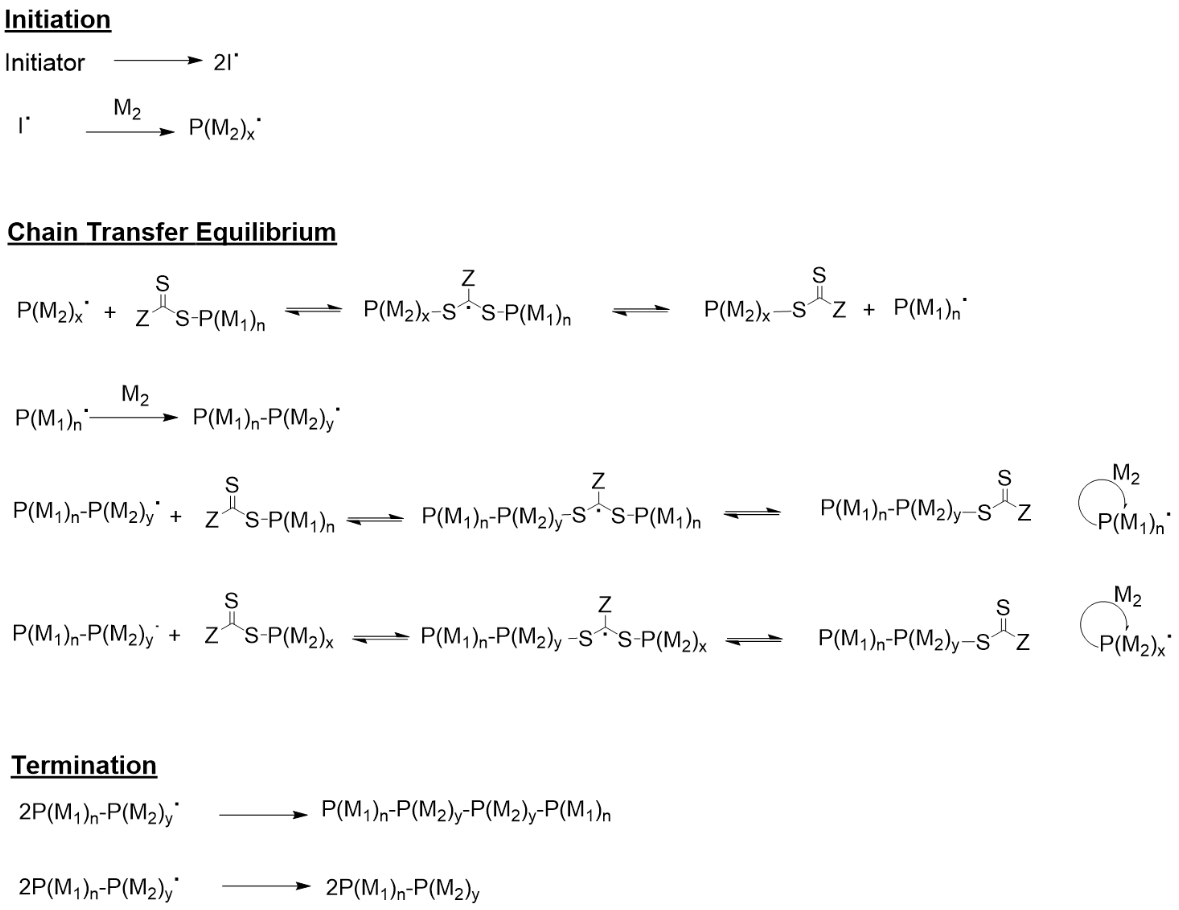

Scheme 1: Reaction mechanism for RAFT diblock copolymerization.

Characterization

High-field nuclear magnetic resonance (H-NMR) spectroscopy

H-NMR spectroscopic analysis was performed using a Bruker Avance III 400 MHz NMR spectrometer. Samples were dissolved in deuterated chloroform and the results were analyzed via MestReNova software. Monomer conversion was determined by comparing the integrated area of vinyl protons of the collected samples with initial stock solution, and using N, N-dimethylformamide (DMF) as the internal standard.

$$Conversion = 1 - \frac{Area_t(vinyl)/Area_t(DMF)}{Area_0(vinyl)/Area_0(DMF)}$$

Where $Area_t(vinyl)$, $Area_t(DMF), Area_0(vinyl), Area_0(DMF)$ are the peak areas associated with the hydrogen associated with vinyl bonds on monomer, and hydrogen associated with methyl groups on DMF at different timeline during (t=t) and at the beginning of an experiment.(t=0).

Classified as Confidential

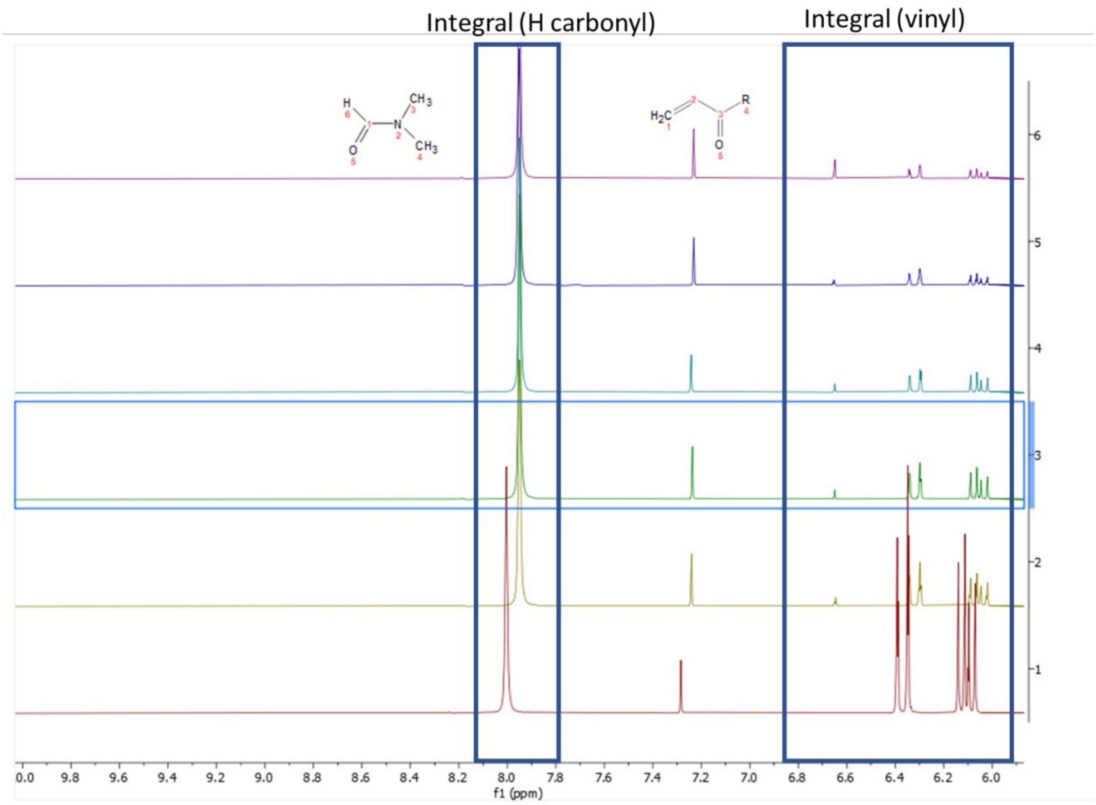

Figure S1: Calculation of monomer conversion from NMR spectra of the samples collected. The peak associated with H bonded to carbonyl group on N, N-Dimethylformamide is used as the internal standard (δ = 7.95ppm), to calculate the change in vinyl peaks (δ = 6.10,6.30,6.70ppm)

Size Exclusion Chromatography (SEC)

DMF

Size Exclusion Chromatography (SEC, also gel permeation chromatography,GPC) was performed on a system comprising a Shimadzu LC-20AT pump, a Shimadzu RID-20A refractive index detector, and an SPD-20A UV–visible detector. The GPC was equipped with a guard column (WAT054415) and 3× Waters GPC columns 2 (WAT044238, WAT044226, WAT044235, 300 mm × 7.8 mm). The eluent was DMF with 10 mM LiBr and eluted at 1 mL min-1 for 45 min in total. The samples were dissolved in DMF with 10 mM LiBr and filtered through 0.20 µm syringe filters. A calibration curve was obtained from poly(methyl methacrylate) (PMMA) standards (Agilent) ranging from 960 to 1 568 000 g mol^{-1}.

THF

The molecular weight and molecular weight distribution of the samples were analyzed on a PSS SECcurity2 GPC system, operated by PSS WinGPC software, equipped with an SDV 5.0 µm guard column (50 x 8 mm), followed by three SDV analytical 5.0 µm columns with varying porosity (1000 Å, 100000 Å and 1000000 Å) (50 x 8 mm) and a differential refractive index detector using THF as the eluent at 40 ˚C with a flow rate of 1 mL·min-1. The GPC system was calibrated using linear narrow polystyrene standards from PSS Laboratories ranging from 682 to 2.52 x 106 g·mol-1 PS (K = 14.1 x 10-5 dL·g-1 and α = 0.70). Molar masses of the PMA samples were determined by universal calibration using Mark-Houwink-Sakurada (MHS) parameters for PMA reported literature values (K = 10.2 x 10-5 dL·g-1 and α = 0.74).

Fourier Transform Infrared (FTIR)

ReactIR (Mettler Toledo) with a 50 ul DS micro flow cell was used to real time inline monitor the concentration of monomer. The equipment was operated by ICIR software, and the sample interval was 10 seconds, after finishing every scan it will automatically generate a CSV file, containing the raw data of the IR spectrum.

Classified as Confidential

Logic for self-optimizing algorithm

After Python program is initiated, user will be prompted to input the number of residence time intervals, and their values, and target monomer conversion to aim for. Timesweep kinetic screening will be carried out automatically and at the end of timesweep experiment, all the IR spectra data will be processed and modelled by the algorithm. The resulting kinetic model will be used to make a T_{res} prediction for a new batch of polymerization based on the target conversion input and the real time conversion will be compared with the target value. If the output value has a discrepancy of ≥2%, the new data, ($T_{res,}$ x) will be appended to the previous dataset for remodelling and a new prediction will be made in the new iteration. Upon target achievement (Δx<2%), the loop will switch to macro-RAFT synthesis mode.

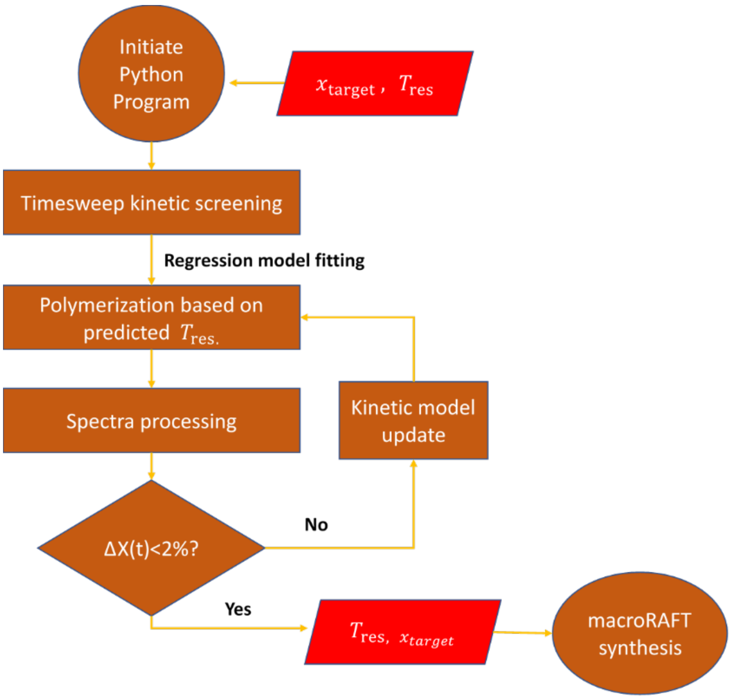

Figure S2: Logic diagram for the algorithm controlling first part of the process flow, which includes timesweep kinetic screening, fine-tuning of the kinetic model and macro-RAFT synthesis.

Supplementary result

Table S5: Monomer conversion under different temperatures, for RAFT polymerization of ethyl acrylate at $[M]_0$=5M, DP_{Target} =50, $[M]_0/[I]_0$=1000. Conversion values were calculated by analysis of vinyl peak on IR, with peak area integrated across 1648-1600cm^{-1}, and compared with values from NMR analysis as the reference.

Temperature/℃	T_{res} /min	X_{IR}	X_{NMR}	*Relative Error*/%
90	10	0.792	0.79	0.25
	20	0.841	0.88	4.43
	30	0.845	0.91	7.14
	40	0.849	0.94	9.68
100	10	0.821	0.88	6.70
	20	0.837	0.92	9.02
	30	0.843	0.93	9.35
	40	0.823	0.87	5.40
110	10	0.827	0.89	7.08
	20	0.828	0.88	5.91
	30	0.832	0.91	8.57

Classified as Confidential

Effect of temperature, initiator concentration and residence time on monomer conversion

Based on equation 6, the strategy to maximize monomer conversion includes increasing initiator concentration, monomer concentration and operating temperature of the reacting system.[2] However, increasing initiator concentration often leads to subsequent decrease in control of the polymerization (broader molar mass distribution of the resulting polymer synthesized) due to increased rate of radical-radical termination. For thermally initiated radical polymerization, the activation energy for k_d is usually much higher than that for k_p and k_t. Therefore, an increase in temperature has a larger impact on initiator decomposition rate than the net propagation rate and this leads to a decrease in monomer conversion (at infinite time).[3] A screening study of monomer to initiator concentration ratio from 125-1000 and temperature from 95-120°C was carried out to find the optimal condition to achieve maximum monomer conversion, without compromising the control of polymerization.

$$\frac{[M]_\infty}{[M]_0} = e^{-2k_p\left(\frac{f[I]_0}{k_t k_d}\right)^{1/2}} \qquad \textbf{(6)}$$

The increase in monomer conversion was not significant when the monomer to initiator concentration ([M]/[I]) ratio was reduced from 1000 to 500, and was more apparent upon further reduction to 125. On the other hand, the dispersity of polymer synthesized showed a negative correlation with [M]/[I] ratio for the argument outlined before. To increase operating temperature, residence time was adjusted accordingly so that it is equal to 5 times the half-life of initiator (AIBN) at the particular temperature, as no further polymerization was expected beyond that, with the absence of newly generated radicals from AIBN. As mentioned by the previous argument, it was found that when operating temperature was increased, the maximum monomer conversion attainable was lower, and the dispersity of polymer synthesized was lower as well, most likely due to lower monomer conversion.

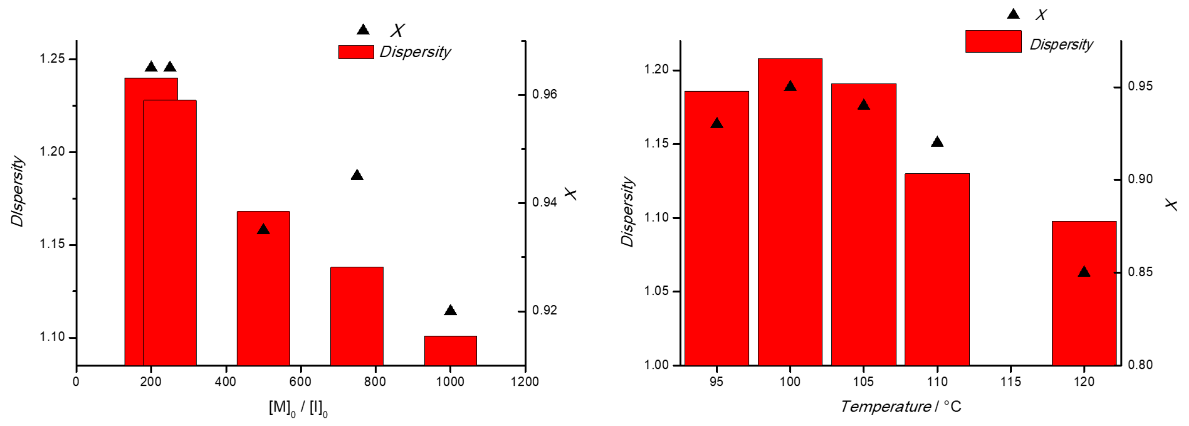

Figure S3: Effect of Initiator concentration (left) and temperature (right) on the dispersity and monomer conversion for homopolymerization of methyl acrylate, mediated by DoPAT, at fixed monomer concentration of 5M and target DP of 100. In the second case study, residence time of polymerization (as indicated on top of each bar plots) is adjusted accordingly to the operating temperature to align with the decomposition rate of initiator in the reacting system.

Effect of macroRAFT molecular weight on the chain extension efficiency

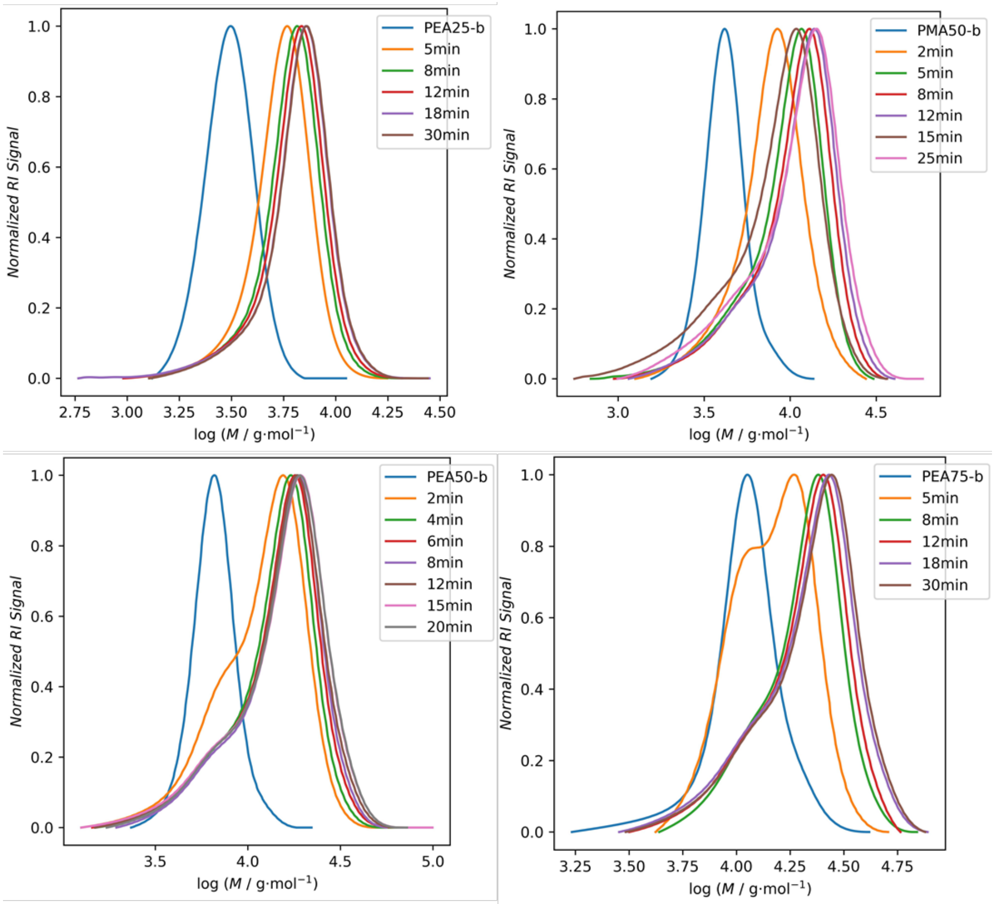

Figure S4: Comparison in the elugrams of 4 sets of diblock copolymers synthesized using macro-RAFT agents of varying molar mass distribution. Anticlockwise starting from top right are: PMA$_{50}$-b-PEHA$_x$,PEA$_{25}$-b-PBA$_x$, PEA$_{50}$-b-PBA$_x$, PEA$_{75}$-b-PBA$_x$.

Classified as Confidential

Type of mixer and influence on the chain-extension

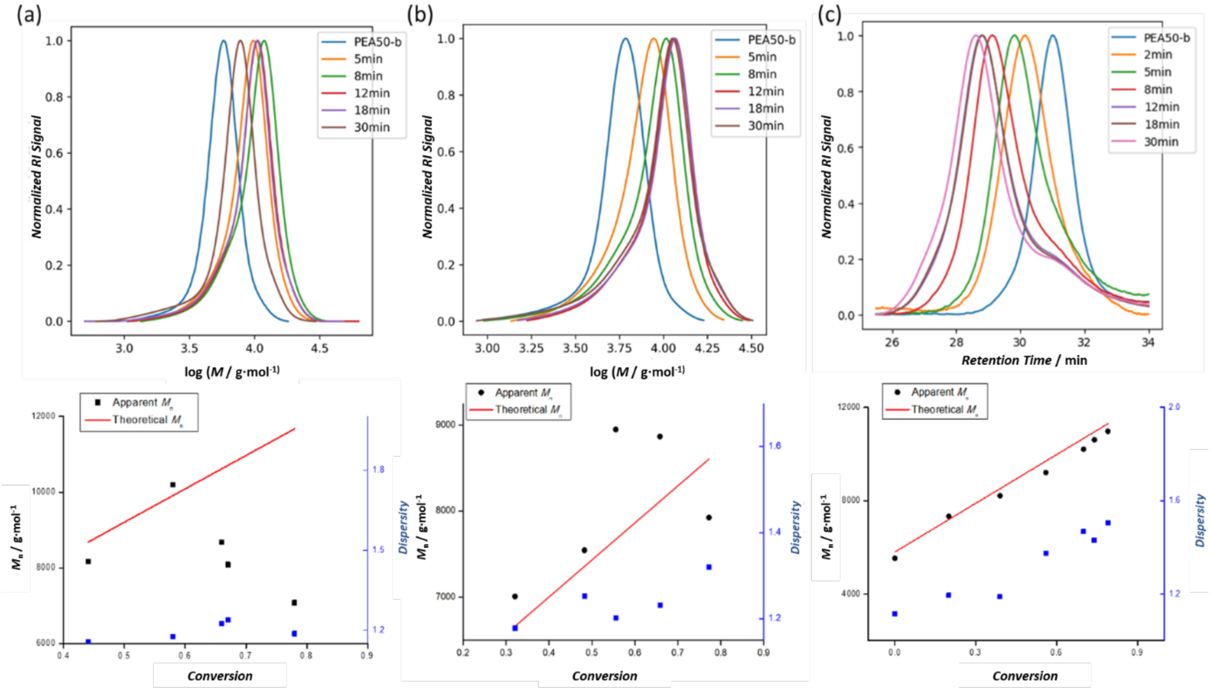

Figure S5: Comparison of MMD for all the di-BCPs synthesized using different type of mixer (a) static micromixer, (b) T-mixer, (c) Y-mixer and prolonger mixing time.

Homopolymerization and diblock Copolymerization with $PEGMEA_{480}$

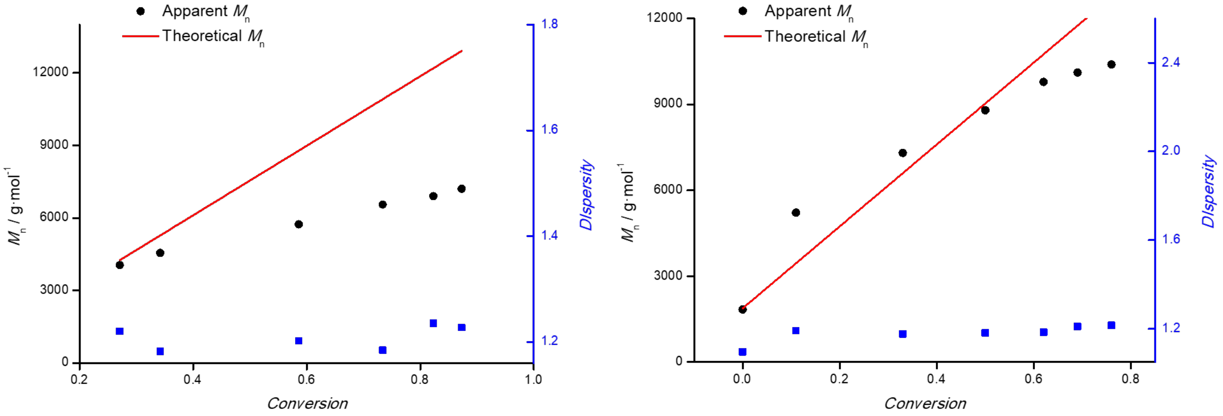

Figure S6: Kinetic data of the synthesized $P(PEGMEA_{480})_{30}$ homopolymer and PEA_{15}-b-$P(PEGMEA_{480})_{30}$ diblock copolymer as a plot of their MMD *vs* conversion.

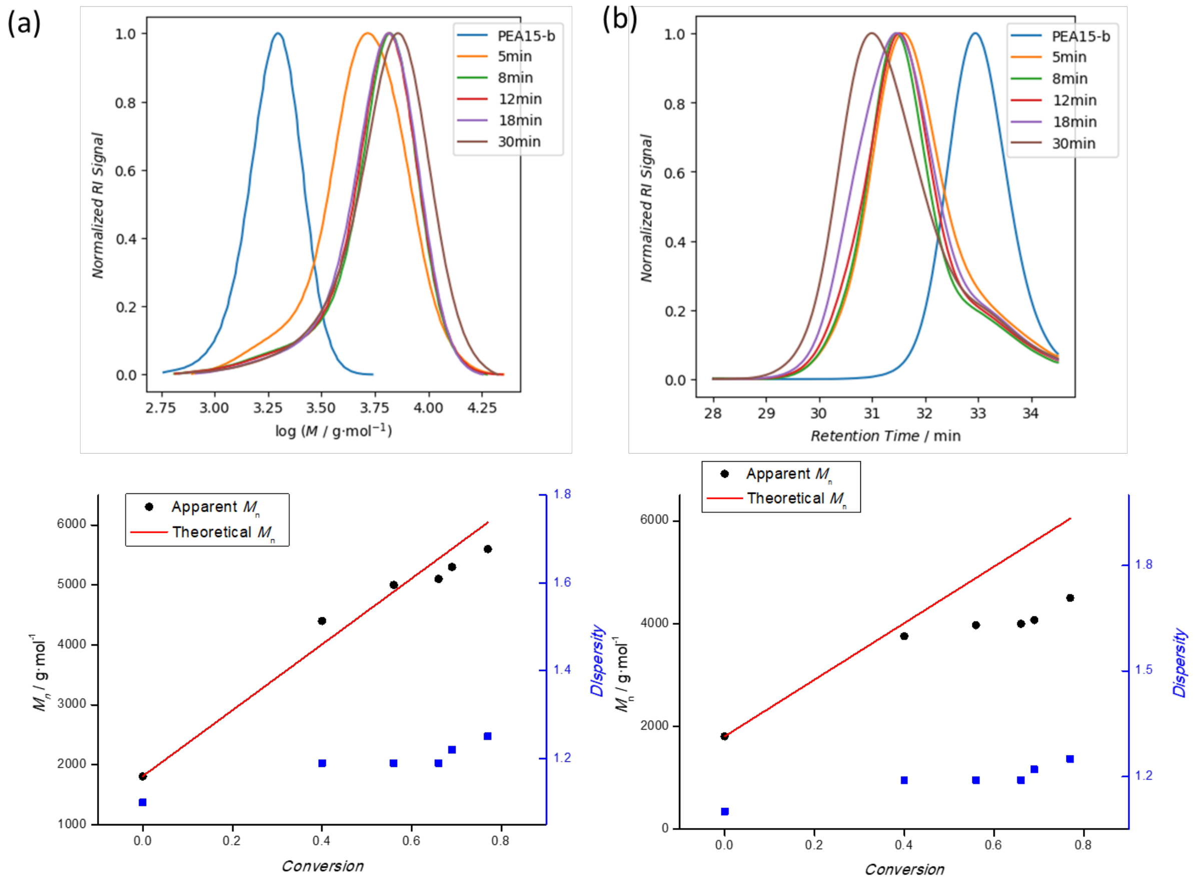

Figure S7: SEC traces of PEA15-b-PEHA30 characterized by (a) THF SEC and (b) DMF SEC and their respective plot of their respective plot in MMD vs conversion (bottom).

Reproducibility study on the synthesis of PEA_{50}-b-PBA_{50}

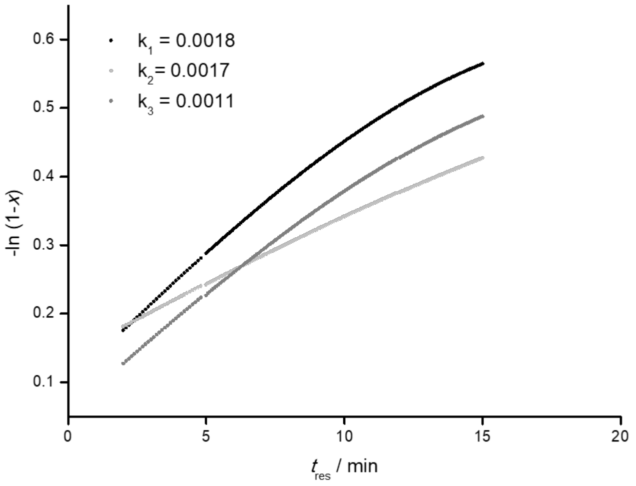

Figure S8 : First order kinetic plots of real-time conversion data collected for three different experiments to synthesize PEA_{50}-b-PBA_{50}.

Three experiments were carried out, on different days and time, to synthesize three sets of PEA50-b-PBA50 diblock copolymer under the exact same reaction condition, and the kinetic profile, individual monomer conversion and molar mass distribution for each of the samples collected were compared to validate the reproducibility of the system. Figure S8 shows the first order kinetic plots for all diblock copolymerization carried out, and their apparent rate constant. The percentage of variance when

comparing the three k is around 8%. Table S6 shows the conversion and molar mass distribution for all the samples collected at different t_{res}, from three sets of experiments. On average, the percentage of variance for X, M_n^{app}, M_W^{app} and $Đ$ are 13.9%, 6.0%, 8.8% and 2.5% respectively. The variance between the three data set is within a reasonable margin, after taking into consideration the errors associated with stock solution preparation, sample preparation for analysis and also fluctuation in background for FTIR analysis, and also limitation in accuracy associated with each of the analytical instrument (NMR and SEC) used. Note that due to the purity issue of AIBN used in these three experiments, their kinetics and maximum monomer conversion are significantly lower than the reported values in the main text (Table 2). However, this is not of concern when we are interpreting the result for reproducibility study since the same source of AIBN was used.

Table S6: Conversion and molar mass distribution of all the samples collected for the synthesis of PEA_{50}-b-PBA_{50}, repeated thrice.

t_{res}	$\boldsymbol{X}$			$M_n^{app}/g \cdot mol^{-1}$			$M_W^{app}/g \cdot mol^{-1}$			$Đ$		
	1	2	3	1	2	3	1	2	3	1	2	3
2	0.143	0.153	0.115	6650	6800	6300	7500	8600	6800	1.12	1.14	1.13
5	0.178	0.238	0.212	7000	7700	6700	8000	8900	7600	1.15	1.15	1.12
8	0.252	0.353	0.284	7100	7900	6900	8200	9300	7700	1.16	1.18	1.13
12	0.298	0.419	0.333	7600	8200	7300	8400	9400	8200	1.12	1.15	1.15
18	0.388	0.43	0.42	7700	8300	7400	8900	9700	8500	1.16	1.27	1.12

References

1. Ferguson, C.J., et al., *Ab Initio Emulsion Polymerization by RAFT-Controlled Self-Assembly.* Macromolecules, 2005. **38**(6): p. 2191-2204.
2. Tobolsky, A.V., *Dead-end Radical Polymerization.* Journal of the American Chemical Society, 1958. **80**(22): p. 5927-5929.
3. Joshi, M.G. and F. Rodriguez, *The kinetics of photoinitiated dead-end polymerization.* Journal of Polymer Science Part A: Polymer Chemistry, 1988. **26**(3): p. 819-826.

Classified as Confidential
